# A new model for the characterization of infection risk in gunshot injuries:Technology, principal consideration and clinical implementation

**DOI:** 10.1186/1746-160X-7-18

**Published:** 2011-10-27

**Authors:** Constantin von See, Majeed Rana, Marcus Stoetzer, Conrad Wilker, Martin Rücker, Nils-Claudius Gellrich

**Affiliations:** 1 Department of Craniomaxillofacial Surgery, Hannover Medical School, Hannover, Germany

**Keywords:** gunshot, infection, basic research, radiology

## Abstract

**Introduction:**

The extent of wound contamination in gunshot injuries is still a topic of controversial debate. The purpose of the present study is to develop a model that illustrates the contamination of wounds with exogenous particles along the bullet path.

**Material and methods:**

To simulate bacteria, radio-opaque barium titanate (3-6 μm in diameter) was atomized in a dust chamber. Full metal jacket or soft point bullets caliber .222 (n = 12, v_0 _= 1096 m/s) were fired through the chamber into a gelatin block directly behind it. After that, the gelatin block underwent multi-slice CT in order to analyze the permanent and temporary wound cavity.

**Results:**

The permanent cavity caused by both types of projectiles showed deposits of barium titanate distributed over the entire bullet path. Full metal jacket bullets left only few traces of barium titanate in the temporary cavity. In contrast, the soft point bullets disintegrated completely, and barium titanate covered the entire wound cavity.

**Discussion:**

Deep penetration of potential exogenous bacteria can be simulated easily and reproducibly with barium titanate particles shot into a gelatin block. Additionally, this procedure permits conclusions to be drawn about the distribution of possible contaminants and thus can yield essential findings in terms of necessary therapeutic procedures.

## Introduction

In addition to complex traumata, gunshot injuries can cause wound infections at the bullet's entrance or exit and within the bullet path. Since the skin as a barrier against bacteria is injured, a wound can fundamentally be assumed to be contaminated with clothing particles, skin bacteria and air bacteria [1]. Current scientific research on possible contaminations along permanent or temporary wound cavities and the resulting surgical recommendations are topics of controversial debate in medical literature [2]. This is not least due to the fact that there is still a lack of clarity about some of the phenomena leading to a temporary wound cavity [3]. Advances in technology are leading to an increase in injuries caused by high-velocity projectiles especially in military conflicts [4]. The temporary wound cavities caused by high-velocity projectiles are significantly wider in diameter, resulting in more extensive tissue destruction [5,6]. The temporary wound cavity is generated both by shock-waves spreading throughout the body prior to the impact of the projectile and subsequent pressure waves spreading within the tissue, which generate a suction effect. Clinical radiological examinations of injuries caused by high-velocity projectiles have shown an increase in the formation of gas cavities in the tissue surrounding the track of the bullet. However, it has not been possible yet to clarify whether those gas cavities are contaminated with exogenous bacteria.

At present, surgeons usually recommend a radical surgical exploration and excision of the affected tissue along the bullet path [7]. The extent of tissue destruction and wound contamination along the bullet path has, however, not been sufficiently analyzed. Most recommendations are therefore based on clinical experience and not on systematic scientific research.

To systematically analyze gunshot injuries, various models illustrating permanent cavity, projectile fragmentation and injuries have been described in literature [8,9]. Forensic gelatin has proved to be the most appropriate material for examining the temporary cavity. The present model provides significant findings in the field of terminal wound ballistics and permits conclusions to be drawn about the surgical procedures required.

Since the tissue removal procedure in bacteriological testing can lead to wrong results or require invasive examination of the specimen, a non-invasive procedure would offer considerable advantages. However, there is no such systematic direct test procedure at present. A specific model simulating bacteria by means of a metal powder which is radio-opaque and permits non-invasive multi-slice CT has therefore been established.

Korac et al. [10] have already used computed tomography (CT) as a non-invasive procedure to analyze different issues using gelatin blocks. Subsequently, other authors have also carried out CT and CBCT scans for clinical and systematic analyses; but the potential for systematic testing offered by gelatin blocks is far from having been fully exploited.

The present paper therefore aims to illustrate wound contamination caused by a variety of high-velocity projectiles in a reproducible and easily presentable manner using gelatin blocks, which are an established instrument in the field of wound ballistics, and to systematically analyze the depth to which bacteria penetrate in different types of gunshot injuries.

## Materials and methods

### Study protocol

The studies were performed using a rifle (Tikka, Riihimäki, Finland) with a barrel length measuring 60 cm. Soft point or full metal jacket bullets of the same weight and comparable kinetic energy (v_0 _= 1096 m/s) were used as ammunition (.222 Winchester).

**Figure 1:**
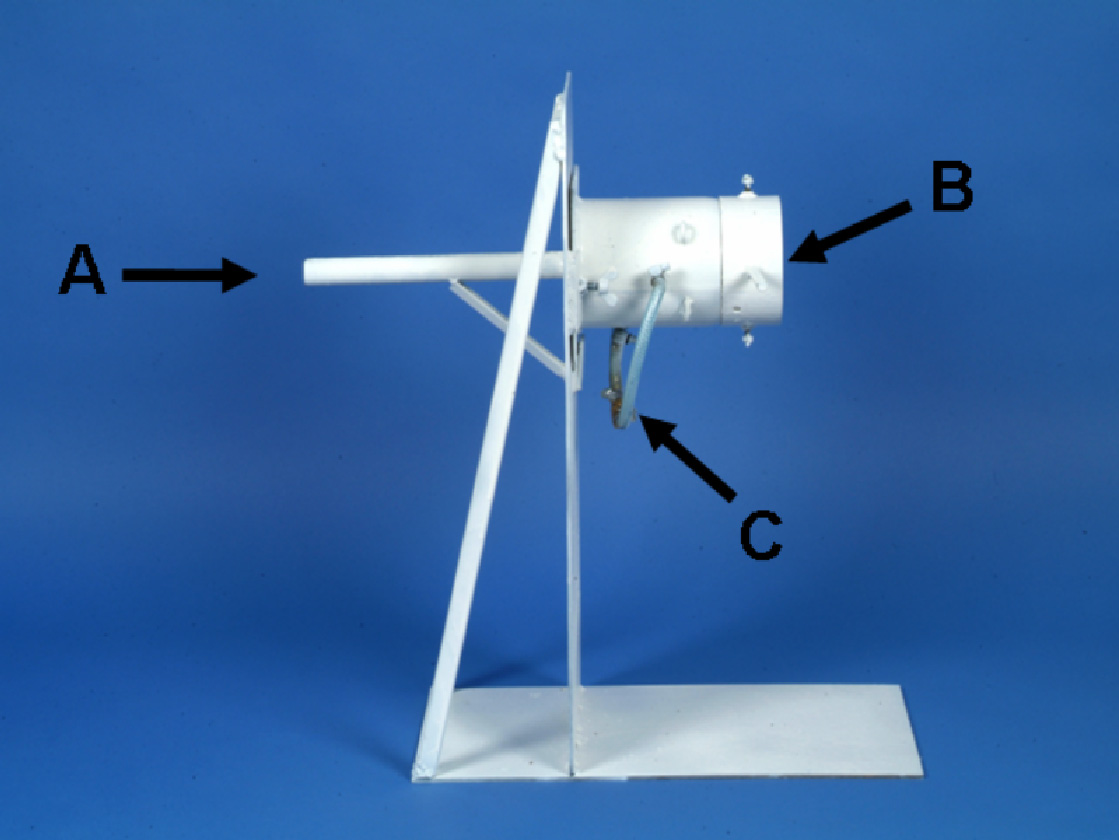
**Schematic assembly of the firing apparatus with the rifle support (A), dust chamber (B) and air pressure valves (C)**.

The tests were conducted with a firing apparatus that included a dust chamber and a rifle support (Figure 1). For each test, 5.0 g barium titanate dust (Aldrich, Steinheim, Germany) with a grain size of 3-6 μm was inserted into the dust chamber. Three air pressure valves, which were linked to an air compressor, were attached to the dust chamber (at the bottom, on the right and on the left). The gelatin blocks were fixed directly behind the dust chamber in the direction of fire.

The tests were performed with gelatin blocks (n = 12). They consisted of 20% porcine gelatin (Merck, Darmstadt, Germany), and water and had an edge length of 12 × 12 × 18 cm.

### Test procedure

One shot was fired into each gelatin block. The gelatin blocks had a temperature of +8-10°C when the shots were fired. They were placed on a support directly in line with the rifle so that the shot passed through the middle of the block. 5.0 g of barium titanate were then distributed in the dust chamber prior to each shot, and a filter paper was inserted to block the dust chamber from the barrel of the rifle. The other end of the dust chamber was directly adjacent to the gelatin block. Shortly before a shot was fired, a momentum-like compressive airpulse of 1.5 bar was applied to the dust chamber that atomized the barium titanate in the chamber. Then the shot was fired from the rifle, which was positioned on its support.

The gelatin blocks were photographed after each shot and multi-slice CT scans were performed for each block (GE Medical Systems, Lightspeed, USA) at 120 kV and 200 mA.

### Analysis

The data obtained were stored in a digital format (DICOM) and transferred to a personal computer for further analysis. Statistical analyses were performed using the Voxim software (Voxim, IVS Solution, Germany). Every 2 cm, a vertical section through the gelatin block was evaluated. After the centre of the gelatin block had been determined, the mean diameter of the permanent cavity was identified. To this end, the length of the permanent cavity towards the centre of the gelatin block was measured radially in eight places, and these eight results were averaged for each vertical section. (Sigma Stat, Version 1.0).

The length of the ruptures was measured analogically in eight places from the centre of the gelatin block for the temporary wound cavity, and the results were averaged for each vertical section. To establish the mean distance between the barium titanate particles deposited within the temporary wound cavity, the infiltration depth was measured from the centre of the gelatin block along the ruptures, and the eight results were averaged for each vertical section.

## Results

Both the gelatin blocks at which shots were fired with a soft point projectile and those at which shots were fired with a full metal jacket projectile were perforated by the projectile or fragments of them.

**Figure 2:**
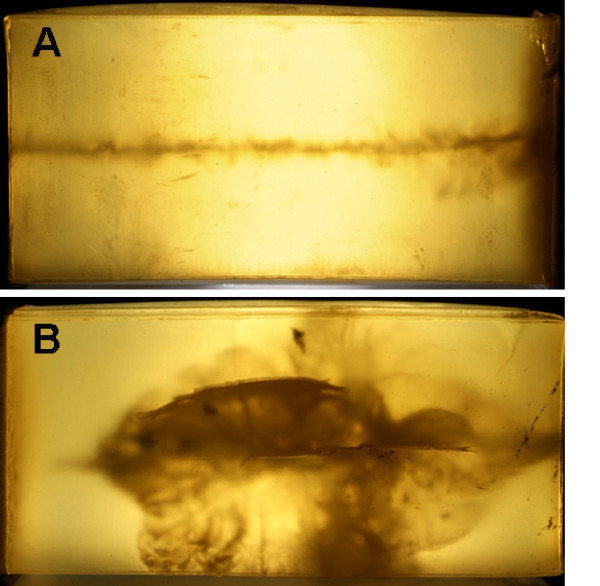
**Photo-optic of the gelatin blocks showing significant differences in the character of the permanent cavity along the bullet path**. The bullets path with full metal jacket bullets did not show any traces of a projectile (A) whereas the gelatin block at which shots were fired with a soft point projectile contained numerous projectile fragments (B).

The photo-optic macroscopic analysis of the gelatin blocks, however, already revealed significant differences in the character of the permanent cavity along the bullet path. The gelatin blocks at which shots were fired with a soft point projectile contained numerous projectile fragments, whereas those at which shots were fired with full metal jacket bullets did not show any traces of a projectile (Figure 2).

### Primary cavitation within the bullet path

**Figure 3:**
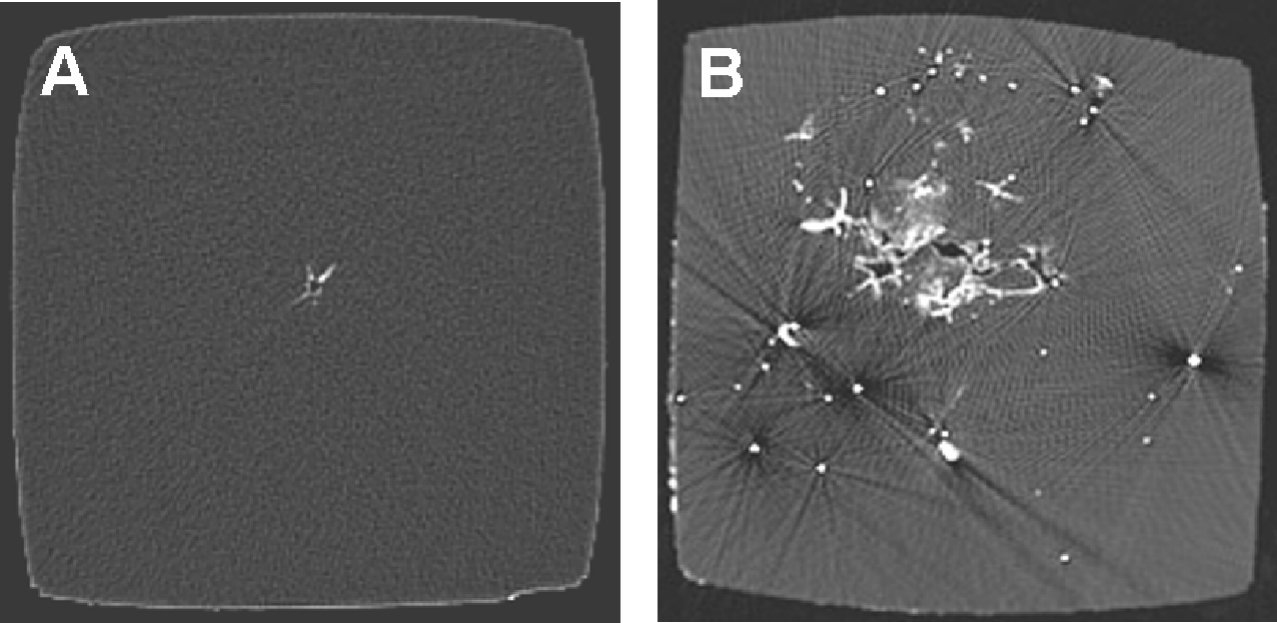
**CT-scans of vertical section through the gelatin block 8 cm from the bullets point of entry**. While in the full metal jacket bullets path (A) only a small permanent cavity with little barium titanate was detectable, the soft point projectile fragmented and lead to a completely different wound characteristics (B).

Soft point and full metal jacket bullets produced cavities of different diameters along the bullet path. Significant differences in the diameter of the cavity between the two projectiles were found 6.0-10.0 cm behind the point of impact of the projectile on the gelatin block. In this area, the gelatin blocks at which shots were fired with soft point bullets showed significantly larger cavities than those at which shots were fired with full metal jacket bullets (Figure 3). Furthermore, numerous projectile fragments could be detected in the gelatin blocks at which shots were fired with soft point bullets.

Irrespective of the cavity diameter or the type of projectile concerned, radio-opaque barium titanate particles appeared in the permanent cavity along the bullet path. The cavity was covered with barium titanate particles along the entire bullet path.

### Analysis of the temporary cavity

To analyze the temporary cavity, ruptures within the gelatin block were investigated radiologically. Both soft point and full metal jacket projectiles produced temporary wound cavities that were significantly wider in diameter (p < 0.05) than the permanent cavities along the entire length of the bullet path within the gelatin block.

An analysis of the diameters of the temporary wound cavities, however, revealed significant differences between the two projectiles examined. The temporary wound cavity reached its maximum size at a penetration depth of 8.0 cm with soft point bullets, whereas that maximum size was reached at a penetration depth of 18.0 cm with full metal jacket bullets.

### Infiltration depth of barium titanate particles in the temporary cavity

The radiological examination of the infiltration depth of barium titanate particles within the ruptures of a temporary cavity in the gelatin block revealed a deposition of particles along the entire bullet path for both types of projectiles examined. In the case of the soft point projectile, there were no significant differences between the size of the temporary cavity and the infiltration depth of the barium titanate particles. In contrast to this, the infiltration depth of barium titanate particles in the case of the full metal jacket projectile was significantly lower in the area 8.0 cm from the entry up to the exit of the projectile as compared with the size of the temporary cavity.

## Discussion

This model for examining potential wound contamination with radio-opaque barium titanate particles is a simple and reproducible method of systematic examination in the field of terminal ballistics. The model permits the infiltration depth of exogenous particles leading to contamination in relation to the bullet path to be analyzed using different projectiles.

Local infections around the bullet path are a frequent complication in gunshot injuries and can lead to more considerable complications, particularly in the long term. Especially in military conflicts, where wound care cannot be administered straightaway because of the tactical situation and elongated evacuation procedures, there is a proportional increase in wound infections [4,11,12]. While clinical examination is primarily focused on the therapeutic approach and wound care [13], the mechanisms of bacterial contamination of gunshot injuries have rarely been investigated.

Depending on the projectiles used, their velocity, the consistency of the tissue penetrated by the bullet etc., extremely different injury patterns appear [14]. To achieve a better understanding of the emerging phenomena, models are used for systematic investigation [15]. There are limits, however, to the extent in which it is possible to apply the results obtained to human tissue, since human tissue has a different elasticity than a gelatin block [16,17].

Materials used for model making behave differently when penetrated by a projectile [18]. Scientific investigations carried out by Rutty et al. showed that the elasticity of forensic gelatin is superior to that of other models (e.g. glycerin soap). Our own investigations also proved that gelatin is partially resilient, which corresponds to clinical experiences.

Previous research carried out to identify the contamination of gunshot injuries focused on providing quantitative proof of the existence of bacteria. It was impossible to determine the relationship to the bullet path. Apart from that, those models are very prone to error and time-consuming. The present model therefore uses barium titanate particles that are comparable in size to bacteria. This permits both a direct evaluation of the barium titanate particles deposited in the gelatin block to be conducted and a comparison with a possible contamination with bacteria to be made. Despite those advantages, the gelatin block does not allow conclusions to be drawn about the reproductive capability of bacteria.

On the other hand, wounding potential is greatly influenced by the projectile's physical characteristics. Projectile construction as well as its material and shape determine the bullet's tendency to deform, fragment or change its flight path upon impact [19].

Although the kinetic energy of both the projectiles tested can be compared, they cause different primary and temporary cavities. This is manifested in different cavity diameters, primarily owing to differences in the depths to which they penetrate into the gelatin block. These can be attributed to the fragmentation behavior of the soft point bullets and the associated higher release of energy over a shorter distance within the gelatin. Those results correspond to those obtained in other studies carried out by Padrta et al., which revealed that destruction projectiles such as soft point bullets caused vaster destruction of tissue than full metal jacket bullets [20].

The gelatin model allows both the permanent and the temporary wound cavities to be examined. The present model shows that particles are transported from the dust chamber into the gelatin block. This corresponds to studies conducted Grosse Perdekamp et al [21], who have already verified the fact that skin bacteria are transported along the bullet path.

The infiltration depth of barium titanate particles largely depends on the projectile used. When soft point bullets are used, the temporary wound cavity is completely covered with barium titanate particles, whereas when full metal jacket bullets are used, it is only partially covered with those particles, and it is smaller in diameter. This can be explained by cavitation effects in connection with particle inertia. This might explain the fact that the suction effect of the negative pressure wave within the temporary cavity also influences the final position of the barium titanate particles. This corresponds to clinical investigations on the distribution of bone fragments after shots have been fired into composite models [22].

## Conclusions

Summing up, it can be concluded that even tissue that is located far from the primary wound cavity can easily be contaminated and damaged by exogenous particles. Depending on the type of projectile used-soft point or full metal jacket-high-velocity projectiles show significant differences as regards the diameter of the permanent or temporary cavity and the degree of contamination with exogenous particles. When soft point bullets are used, both temporary and permanent wound cavities must be expected to be contaminated completely, whereas when full metal jacket bullets are used, it can be assumed that they will only be partially contaminated with exogenous particles is to be assumed.

Thus, the present model for the first time allows a rapid and easy analysis of contamination with exogenous particles in gunshot injuries of different ballistic properties in relation to the bullet path.

## Competing interests

The authors declare that they have no competing interests.

## Authors' contributions

CS, MR, MS, CW, MRu, and NCG conceived of the work and participated in its design and coordination. CS and MR made substantial contributions to data acquisation and conception of manuscript. CS and MR drafted and designed the manuscript. CW, MRu, NCG have been involved in drafting the manuscript. NCG was involved in revising the manuscript. All authors read and approved the final manuscript.
